# Medication-Related Osteonecrosis of the Jaw (MRONJ) due to Simvastatin: An Unusual Case Report

**DOI:** 10.29252/wjps.10.1.132

**Published:** 2021-01

**Authors:** Sahand Samieirad, Ali Labafchi, Khashyar Famili, Haleh Hashemzadeh

**Affiliations:** 1 Oral and maxillofacial Diseases Research Center, Mashhad University of Medical Sciences, Mashhad, Iran; 2 Student Research Committee, Faculty of Dentistry, Mashhad University of Medical Sciences, Mashhad, Iran; 3 Department of Oral and Maxillofacial Surgery, Dental School, Mashhad University of Medical Science, Mashhad, Iran; 4 Department of Orthodontics, Tehran University of Medical Science, Tehran, Iran

**Keywords:** Bisphosphonate-related, Osteonecrosis of the jaw, Simvastatin

## Abstract

Medication-related osteonecrosis of the jaw (MRONJ) is a serious pathological condition that usually results from anti-resorptive or anti-angiogenic drugs. We aimed to report an unusual MRONJ in a female patient due to long-term simvastatin administration. A 48-year female was referred to the Department of Oral and Maxillofacial, Mashhad Dental School, Mashhad, Iran in Dec 2019. She complained of pain, swelling, and infection in the right mandibular area with a history of extraction. Based on medical history, the patient received 40 mg of simvastatin daily for ten years to control hypercholesterolemia. According to clinical and radiographic examinations, as well as previous medical and dental records, the lesion diagnosis was detected as MRONJ. Moreover, histopathological examination of the lesion confirmed our clinical diagnosis. The necrotic bone was removed with caution. The PRF was then inserted, and the flap was sutured without any tension. No complications were observed on following-up, and all symptoms were discontinued. There was a correlation between the administration of high-dose simvastatin and MRONJ. Moreover, more clinical investigation with larger sample sizes is suggested.

## INTRODUCTION

Although, medication-related osteonecrosis of the jaw (MRONJ) is a rare condition, the known adverse effect of anti-resorptive or anti-angiogenic drugs. ^1^^, ^^2^ MRONJ manifests itself as the necrotic exposed bone lesions, generally in the mandible, and remain for at least eight weeks. ^2^^-^^4^ This condition may occur following tooth extraction or other types of oral surgeries. Osteonecrosis of the jaw (ONJ) has also been associated with new medications. ^1^^-^^5^

Regarding the literature, there is no effective therapy for the treatment of ONJ at the moment. Hence, to avoid new cases of MRONJ, it is important for all oral and maxillofacial surgeons to be completely up-to-date with the etiopathological aspects of this lesion and to be aware of those drugs considered a danger. ^1^^-^^5^

We aimed to report an unusual MRONJ case due to long-term simvastatin administration in the posterior mandible of a female patient.

## CASE REPORT

A 48-year-old non-smoker female was referred to the Department of Oral and Maxillofacial, Mashhad Dental School, Mashhad, Iran, in Dec 2019. 

**Fig. 1:**
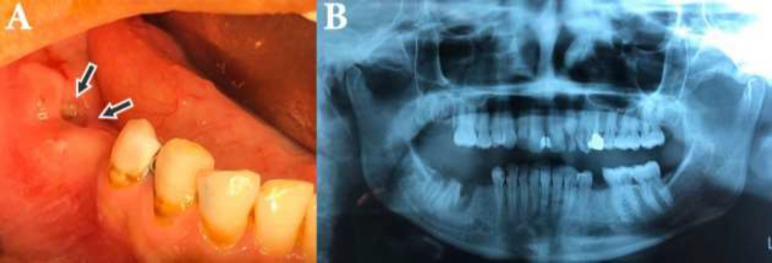
Clinical (a) and radiographic (b) view of MRONJ

The patient complained of swelling, pain and purulent drainage in posterior right mandible two months ago (Figure 1A). The panoramic radiograph (OPG) showed the segment of the necrotic bone within the ill-defined radiolucency (Figure 1B). The patient complained of painful right mandibular molars traumatically extracted about three months earlier (teeth 46 and 47). 

The patient had no history of radiotherapy to the maxillofacial region as well as steroid therapy and malnutrition. Moreover, the bacterial culture test results of the region were negative. Therefore, we ruled out the probability of osteomyelitis, osteoradionecrosis, and steroid-induced necrosis. According to the past medical history, the patient was treated with 40 mg of simvastatin daily for 10 years to control hypercholesterolemia. She did not receive any bisphosphonate drugs.

The possible diagnosis was MRONJ due to simvastatin drug, given the clinical and radiographic examinations as well as previous medical and dental records.

**Fig. 2:**
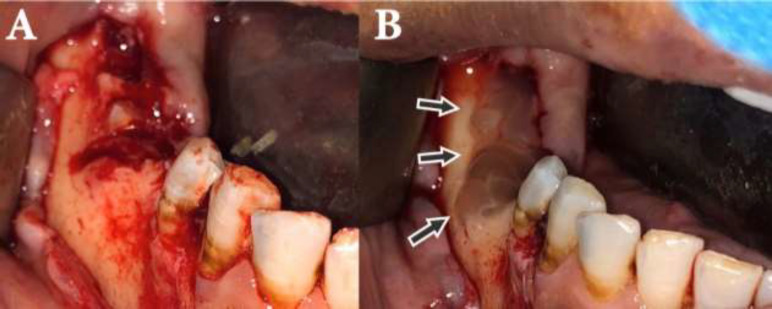
A: Full-thickness flap elevation showed necrotic segment, B: PRF was adapted to the exposed bone region

After obtaining the informed consent from the patient, the surgeon decided to perform the debridement and sequestrectomy of the necrotic mandibular lesion under local anesthesia. Moreover, PRF (platelet-rich fibrin) and the buccal flap were used for mandibular lesion coverage (Figure 2). Histopathological examination of the lesion confirmed our clinical diagnosis. The healing of the lesion was uneventful, with no complication. 

## ETHICAL APPROVAL

All procedures performed in this study involving the human participant were following the ethical standards of our institutional Ethics Committee, Mashhad University of Medical Sciences, Mashhad, Iran (IR.MUMS.REC.1399.385) and in accordance with the 1964 Helsinki declaration. Informed consent was taken from the patient. 

## DISCUSSION

Bisphosphonates (BPs) and other antiresorptive medicines block the function of osteoclast and increase apoptosis, which contributing to reduced bone resorption and remodeling. ^6^^, ^^7^ Based on the recent evidence, taking the high dose or long-term use of oral and IV forms of BP may result in adverse effects such as bisphosphonate-related osteonecrosis of the jaw (BRONJ). ^2^^, ^^4^^, ^^8^

This condition is also associated with other medications including antiresorptive agents, denosumab and some antiangiogenics such as sunitinib and bevacizumab. ^2^^, ^^4^ Hence, in the literature, the term BRONJ was eventually replaced by MRONJ. ^1^^-^^5^

Simvastatin is a cholesterol-lowering drug with some therapeutic effects on bone angiogenesis. ^9^ It appears to play a significant role in bone regeneration by directly participating in osteoblast activation as well as osteoclast inhibition. ^9^ Statins drugs inhibit the mevalonate pathway by inhibiting HMG-CoA reductase. ^10^ In other words, both BPs and simvastatin can block the mevalonate pathway and have a similar effect on osteoclasts, even though they impact different enzymes. ^5^^, ^^9^^, ^^10^

Long-term use of simvastatin might cause osteonecrosis of the jaw, similar to the present case. Two cases of maxillary MRONJ were reported in healthy and non-smoker patients who undertook high-doses of simvastatin for 20 years. ^4^

## CONCLUSION

There was a correlation between simvastatin and MRONJ. More clinical investigation with larger sample sizes is suggested.
